# Antioxidative Property and Molecular Mechanisms Underlying Geniposide-Mediated Therapeutic Effects in Diabetes Mellitus and Cardiovascular Disease

**DOI:** 10.1155/2019/7480512

**Published:** 2019-04-04

**Authors:** Ning Li, Ling Li, Haiming Wu, Heng Zhou

**Affiliations:** ^1^Department of Cardiology, Renmin Hospital of Wuhan University, Wuhan 430060, China; ^2^Cardiovascular Research Institute, Wuhan University, Wuhan 430060, China; ^3^Hubei Key Laboratory of Cardiology, Wuhan 430060, China; ^4^Institute of Hepatobiliary Diseases of Wuhan University, Zhongnan Hospital of Wuhan University Hubei Key Laboratory of Medical Technology on Transplantation, Wuhan, Hubei 430071, China

## Abstract

Geniposide, an iridoid glucoside, is a major component in the fruit of *Gardenia jasminoides* Ellis (Gardenia fruits). Geniposide has been experimentally proved to possess multiple pharmacological actions involving antioxidative stress, anti-inflammatory, antiapoptosis, antiangiogenesis, antiendoplasmic reticulum stress (ERS), etc. *In vitro and in vivo* studies have further identified the value of geniposide in a spectrum of preclinical models of diabetes mellitus (DM) and cardiovascular disorders. The antioxidative property of geniposide should be attributed to the result of either the inhibition of numerous pathological processes or the activation of various proteins associated with cell survival or a combination of both. In this review, we will summarize the available knowledge on the antioxidative property and protective effects of geniposide in DM and cardiovascular disease in the literature and discuss antioxidant mechanisms as well as its potential applications in clinic.

## 1. Introduction

**Figure 1:**
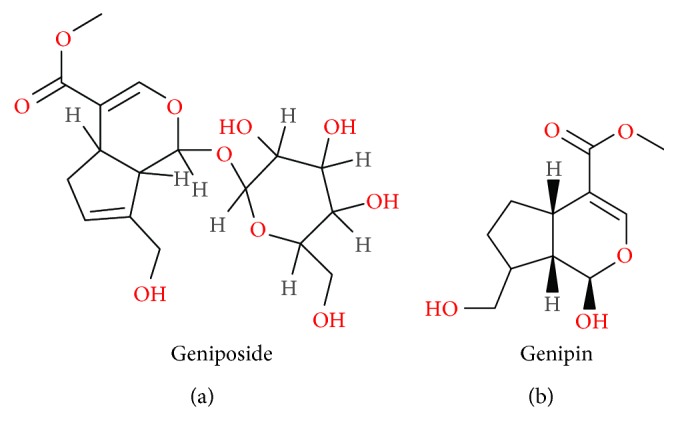
Chemical structure of geniposide (a) and genipin (b).

In the early stage of modern medicine development, biologically active compounds extracted from plants have played a crucial role in providing medicines to combat diseases. Medicines extracted from plants continue to account for a significant niche in the treatment of diseases all over the world [1]. Naturally derived compounds have fewer reported advice effects than allopathic medicine, indicating that it could be safer to use for a longer period of time. Geniposide (Figure 1(a)) is one such plant-derived medicine that has been traditionally used as a folk medicine for hundreds of years in Asian countries.

Geniposide is one of the natural components extracted with ethanol from the fruit of *Gardenia jasminoides* Ellis, the concentration of which in *Gardenia jasminoides* Ellis is 73.44 ± 2.62 mg/g. As a major iridoid glycoside and glucagon-like peptide-1 (GLP-1) receptor agonist, geniposide is supposed to exert various biological effects of the herbs [2–4]. Genipin (Figure 1(b)) is the primary metabolite and aglycon of geniposide. Previous studies have revealed that genipin is transformed from geniposide by the intestinal microflora enzymes in the bowel, which indicates that the major form of geniposide in circulating blood may be genipin [5–7]. Until now, geniposide has been used extensively for the reason that it rarely causes severe toxic effects [8]; meanwhile, it has various pharmacological properties including antioxidative stress [9], anti-inflammatory [5], antiapoptosis [10], antithrombus [11], antiendoplasmic reticulum stress [12], and antifungi [13]. Geniposide exhibits a number of merits as a promising therapeutic agent due to its properties and is currently in preclinical models for a variety of conditions like neurological disorders [14], liver diseases [15], diabetes mellitus (DM) [16], cardiovascular disorders [17], tumors [18], and other conditions.

DM and cardiovascular disease are growing at epidemic proportions giving rise to heavy social, economic, and health burden. Although many efforts and advances have been made, the current therapeutic approaches for the treatment of DM and cardiovascular disease are still far from satisfactory, principally because of serious adverse effects [19]. Hence, some novel therapeutic candidates are now continually being explored to obtain better outcomes. In the last decades, a considerable attention has been paid on functional properties of natural compounds derived from plants for their potential therapeutic purposes. The antioxidative property and potential effects in DM and cardiovascular disease of geniposide make it become an attractive candidate for the treatment, particularly in type2 DM and cardiomyopathy. In this review, we aim to highlight relevant experimental researches performed in terms of pharmacokinetic properties, the antioxidative property, and therapeutic effects of geniposide in DM and cardiovascular disease.

## 2. Pharmacokinetics

Extensive studies to elucidate the pharmacokinetic properties of geniposide have been performed on different species including rats and humans. Here, we will illustrate its properties according to absorption, distribution, metabolism, and excretion.

**Figure 2:**
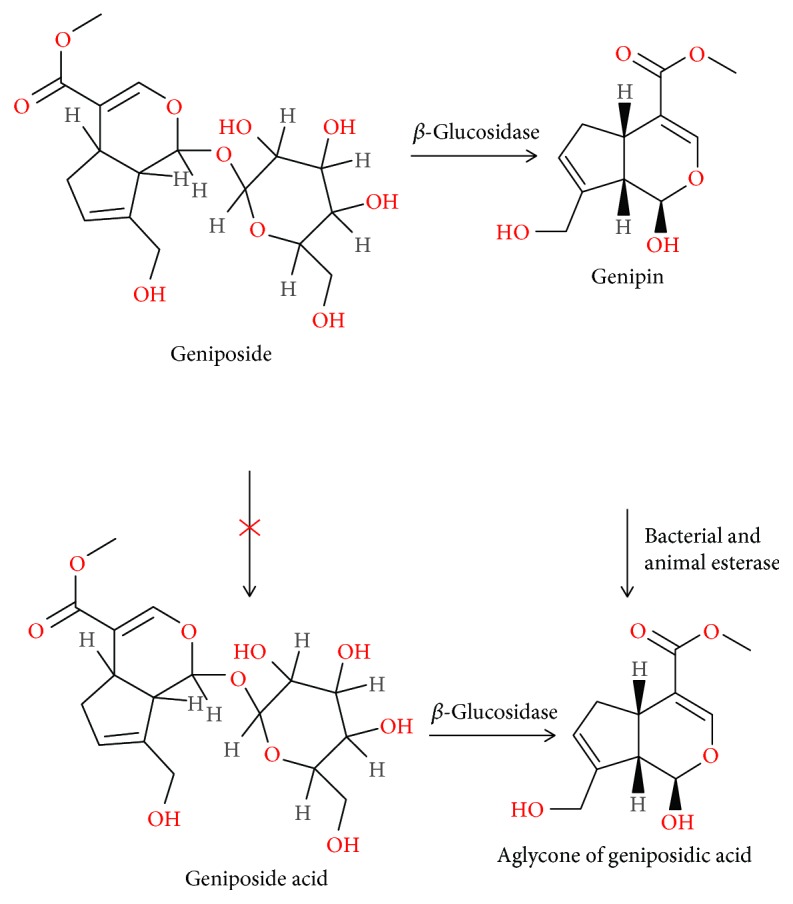
Schematic diagram of the metabolic process of geniposide by *β*-glucosidase and esterase [2].

Geniposide exhibited excellent choleretic effects when it was administrated orally or intraduodenally. However, this effect was blocked when injected intraportally. By contrast, genipin, which is the aglycone of geniposide, possessed favorable choleretic effects by either intraportal or intraduodenal administration. Furthermore, *in vitro and in vivo* studies have demonstrated that geniposide could be transformed to genipin by the intestinal microflora enzymes (*β*-glucosidase) in the bowel. This indicates that geniposide has no activity as a cholagogue by itself unless it is transformed to another active metabolite, genipin. In the presence of ammonia, genipin could be further transformed to a new nitrogen-containing compound, genipinine. Despite that both geniposide and genipin contain methyl ester, which seem to be hydrolyzed to genoposidic acid, only genipin in fact could be hydrolyzed to the aglycone of genoposidic acid by bacterial and animal esterase [20]. A total of 24 species of human bacteria in intestinal tract could help geniposide transform into genipin (Table 1). Although intestinal bacteria could produce five types of *β*-glucosidase, it seems that only one fraction from *Eubacterium* sp. A-44 and *Ruminococcus* sp. PO1-3 is able to hydrolyze geniposide to genipin. Notably, esterase from *Eubacterium* sp. A-44 as well as pig liver could not hydrolyze geniposide to geniposide acid directly, but could hydrolyze genipin to geniposide acid [21].  Figure 2 gives a schematic diagram of metabolic progress of geniposide by *β*-glucosidase and esterase. Yu et al. [22] discovered that the stability of geniposide in different pH K-R solutions did not alter, which revealed that the acidic conditions may have no direct influence on the stability of geniposide. Geniposide was not stable in duodenum perfusions but stable in jejunum, ileum, and colon perfusions, the difference of which may be caused by the intestinal contents. However, after adjusting the pH values of perfusions to 7.0, geniposide was observed to be stable in different regions of the intestine. These two phenomena indicate that the acidic conditions may affect geniposide indirectly through affecting the intestinal contents but not influence the stability of geniposide directly. Adding verapamil, one of the well-known inhibitors of P-glycoprotein (P-gp) could significantly elevate the absorption of geniposide 2.4-fold, but the absorption was not changed by EDTA, one of the typical inhibitors of cell membrane transport channels, which hinted that the transportation of geniposide might be associated with P-gp rather than membrane transport channels. Intriguingly, another traditional medicine, notoginsenoside R_1_, at 0.1 and 0.2 mg/mL concentrations was able to significantly enhance the intestinal absorption of geniposide (1.424 mg/mL) by 1.7- and 1.4-fold in a fashion similar to that of a P-gp inhibitor [23]. Likewise, P-gp is also the efflux transport of genipin. And the intestinal absorption rate and permeability value of genipin were dramatically enhanced by the genipin/hydroxypropyl-*β*-cyclodextrin inclusion complex owing to the solubilizing effect and P-gp inhibitory effect of hydroxypropyl-*β*-cyclodextrin [24].

Using a high-performance liquid chromatography (HPLC) with a column-switching system, the simultaneous concentration of geniposide and genipin in mouse plasma after oral administration of 1200 mg/kg hot-water extract Gardenia fruits (1200 mg extract contained 216.4 mg geniposide, while the genipin in extract was a trace amount) was detected after 15 min. The results demonstrated that the concentration of geniposide in plasma peaked (approximately 103.1 *μ*g/mL) at 30 min and reduced gradually. In contrast, the amount of genipin was too small to detect, which seemed to reach a peak (approximate 0.07 *μ*g/mL) at 60 min. This suggested that a large amount of geniposide went into the circulating blood while only a very small amount of genipin could go into the blood after being hydrolyzed for the reason that genipin was so labile a substance that it could easily react with proteins and amino acids [25]. In particular, the oral bioavailability of geniposide in rats was obviously improved when *Gardenia jasminoides* Ellis was combined with *Cortex magnoliae officinalis* and *Fructus aurantii immaturus*. Because of the herb-herb interaction, the pharmacokinetic process of geniposide from Chinese medicinal formula was more complicated and unpredicted. Hence, geniposide in some certain formula should be paid closer attention compared with its single use [26].

In a model of anesthetized rats with intravenous administration [27], geniposide displayed a dose-dependent manner in circulating blood within the dosage range of 10-100 mg/kg. In the dosage of 10 mg/kg, the area under concentration-time curve (AUC) of geniposide in circulating blood was significantly smaller than that in bile, indicating that geniposide was mainly excreted via hepatoenteral circulation. Intriguingly, acupuncture treatment of specific acupuncture points for the liver and bladder channels had no obvious influence on the pharmacokinetics of geniposide in rat blood, bile, and liver although cumulative studies showed that acupuncture or electroacupuncture treatments may interact with drugs in terms of pharmacokinetics [28]. Furthermore, the distribution of geniposide in rats was also studied. After oral administration of geniposide, the maximum concentration (Cmax) emerged at 30 min in the spleen and liver, at 120 min in the kidney and brain. Among all the tissues and organs investigated, kidney was the organ which possessed the highest concentration of geniposide. The AUC_0→4h_ values after the administration of geniposide were according to the order of brain <lung<heart<liver<spleen<kidney [29].

The metabolic process of geniposide is complicated and remains unclear. Using online liquid chromatography-mass spectrometry (LC-MS) data acquisition and off-line data processing methods involving extracted ion chromatogram, multiple mass defect filters, fragment ion searching, and isotope pattern filtering, a total of thirty-three different metabolites of geniposide were captured in the rat liver, urine, and plasma. Among these metabolites, six were in rat heart, twelve in liver, six in brain, six in lung, three in spleen, twelve in kidney, and four in rat liver microsomes. However, only six of them may exert pharmacological actions. The corresponding metabolic reactions of geniposide involved hydroxylation, hydrolysis, taurine conjugation, demethylation, hydrogenation, decarboxylation, cysteine S-conjugation dehydration, methylation, glucosylation, sulfate conjugation, and their composite reactions [24]. Additionally, the metabolism of geniposide in immune tissues of rats with adjuvant arthritis was also investigated. Rats with adjuvant arthritis were divided into three groups and given geniposide intragastrically at 120, 66, and 33 mg/kg, respectively. Subsequently, the biotransformation of geniposide in different organs was detected and a total of four major metabolites were identified, namely G1, G2, G3, and G4. The distribution of geniposide and its metabolites is shown in Table 2 [26]. Taking plasma for example, after oral administration of geniposide, the parameters in pharmacokinetics of rats with or without adjuvant arthritis exhibited great differences. AUC_0→∞_, the mean residence time (MRT), the clearance from the total body (CL), and the apparent volume of distribution in adjuvant arthritis groups were significantly higher than those in the normal group, suggesting that the pharmacokinetics of geniposide was altered under the condition of adjuvant arthritis [30].

Apart from rapid absorption into the circulating blood, wide distribution in tissues and organs, and complicated metabolism, geniposide could be also eliminated rapidly from the plasma within 12 h in rats [29]. Besides, the excretion of geniposide in the human body was also investigated. The Naoxuening injection (its major component is geniposide) was administrated intravenously, and the concentration of geniposide in urine was detected at various points in time. The results showed that geniposide was primarily excreted in the original form via the kidney. At 2 h, 4 h, and 10 h, the excretion level accounted for 52%, 63%, and over 90% of the initiative dosage [31].

Taken together, the pharmacokinetics of geniposide is still at the groping stage. Different experimental subjects, different administration routes, and different disease models may contribute to completely different outcomes. Geniposide predominately exists in the liver and kidney and is excreted in the original form via the kidney, which provides reliable evidence for its dosage regimen and pharmaceutics.

## 3. Effects of Geniposide on Cytochrome P450 and P-Glycoprotein

Although traditional medicines have become increasingly general all over the world, there are still some crucial possibilities for interaction between these traditional medicines and other allopathic medicines, which tends to steal the spotlight. A great many studies have uncovered that geniposide and genipin could affect the activities of enzymatic proteins and transporters in intestines and liver. Thus, the absorption and degradation of some certain drugs may give rise to altered plasma concentration of these drugs. For example, genipin has been proved to modulate the activity of cytochrome P450 (CYP450) 3A monooxygenase and P-gp [32]. CYP450 isoenzymes, which are mainly distributed in the liver and some extrahepatic tissues, play vital roles in the metabolism of drug oxidation reaction [33]. Inhibiting CYP450 isoenzymes may lead to target organ toxicity because they could increase exposure of these affected drugs. Therefore, the induction of xenobiotics to CYP450 isoenzymes is one of the main causes of therapeutic failure [34]. In human hepatoma HepG2 cells, genipin remarkably inhibited the level of mRNA and protein of CYP3A4 as well as CYP2C19, but significantly induced the expression of CYP2D6. To our knowledge, some traditional Chinese medicines involving *Rhizoma coptidis* [35] and *Lotus* leaf [36] have been shown to act as strong inhibitors of CYP2D6 [32]. This signifies that the target organ toxicity will be reduced and therapeutic effects will be enhanced when *Gardenia jasminoides* is combined with *Rhizoma coptidis* or *Lotus* leaf in traditional Chinese formulae. On the other hand, the metabolism of CYP2D6 could inhibit the pharmaceutical activity of some certain ingredients in traditional medicine, such as aconitine [37]. Similarly, in a rat model of nonalcoholic steatohepatitis, geniposide remarkably suppressed the expression of CYP2E1 [38]. Hence, we speculate that the combination with *Gardenia jasminoides* could expedite the degradation of aconitine and reduce its availability.

P-gp, one of the very famous members in the superfamily of ATP-binding cassette (ABC) transporters, was first characterized in multidrug-resistant Chinese hamster ovary (CHO) cells. P-gp is mainly coded by multidrug resistance gene 1 (MDR1) and plays a crucial part in eliminating xenotoxins against steep concentration gradients [39–41]. P-gp is widely distributed in various tissues and cells including the blood-brain barrier, intestinal epithelium, and tumor cells. The potential functions of P-gp in intestinal absorption as well as prehepatic elimination of a considerable amount of drugs have also been figured out [42, 43]. In doxorubicin-resistant cells, geniposide (50-100 *μ*M) in a dose-dependent manner reversed P-gp-mediated MDR by inhibiting the expression of P-gp and its transport function [44]. Genipin has been also disclosed to be a substrate of P-gp and interact with the drug-binding site of P-gp competitively. Further study showed that the induction of genipin on P-gp by genipin is for the reason that it could strongly elevate the expression of MDR1 protein in hepatoma HepG2 cells via activating the basal P-gp ATPase [32]. From our perspective, the clinical application of genipin or geniposide with other drugs should be paid special attention, meantime the P-gp blockers should be also considered appropriately for the sake of the drug efficiency.

## 4. Toxicology

At the beginning, Yamano et al. disclosed that the crude extract of gardenia fruit, which was originally used as a kind of dye, could lead to hepatotoxicity in rats in both a time-dependent and dose-dependent manner after acute oral exposure [45]. Notably, intraperitoneal injection of geniposide did not trigger hepatotoxicity in rats but oral administration did. And oral administration of geniposide at a dose of 320 mg/kg or intraperitoneal injection of genipin at a dose of 80 mg/kg had similar effects, both of which could increase the activity of alanine transaminase (ALT) and aspartate aminotransferase (AST). In particular, the geniposide-induced hepatotoxicity was completely suppressed by chloramphenicol, indicating that chloramphenicol may block the hydrolysis of geniposide to genipin via modulating the microenvironment of intestinal microflora. These phenomena proved that it was genipin, not geniposide, that was likely to cause hepatotoxicity [46]. An acute toxicity study showed that the median lethal dose (LD50) of geniposide was 1431.1 mg/kg in rats. When the dose of geniposide in rats was over 574 mg/kg, hepatotoxicity could be triggered and the toxic effect usually emerged at 1-2 days after oral administration. A subchronic toxicity study on rats suggested that oral administration of geniposide at a normal dose of 24.3 mg/kg or less even in the repeated dosing for three months would not give rise to hepatotoxicity [47]. Another subchronic toxicity study revealed that 3-month ingestion of geniposide intake (60 mg/kg/day) did not cause any severe liver and kidney injury in rats [8]. However, in a 13-week toxicity study, some hematological parameters, the percentage of reticulocytes (Retic %), and the weight of some relative organs in male rats administered geniposide (100 mg/kg) significantly increased. In a 26-week toxicity study, the level of ALT, AST, electrolyte including potassium (K^+^) and sodium (Na^+^), red blood cell (RBC), white blood cell (WBC), haemoglobin (HGB) levels, Retic %, and the weight of liver and spleen in male rats also significantly increased [48]. The acute hepatotoxicity and nephrotoxicity in both jaundice rats induced by *α*-naphthylisothiocyanate and healthy rats were also evaluated. Geniposide at the dose of 120 mg/kg could lead to acute liver and kidney injury in rats and displayed a time-toxicity relationship [49]. Compared with the healthy group, the levels of ALT, AST, alkaline phosphatase (ALP), blood urea nitrogen, total bilirubin, and creatinine activity in rats administrated geniposide at the dose of 1.2 g/kg were statistically elevated at 48 h and 72 h [50]. In a rat model after intragastric administration of geniposide (300 mg/kg) for three days, ALT, AST, *γ*-glutamyltransferase (*γ*-GT), ALP, and total bile acids in serum were significantly elevated. Meanwhile, geniposide (300 mg/kg) also inhibited the expression of small heterodimer partner (SHP), farnesoid X receptor (FXR), and bile salt export pump (BSEP), suggesting that the dose-related liver injury of geniposide may be related with the abnormal expression of bile acid–regulating genes.

The mechanisms of hepatotoxicity from geniposide may be involved in oxidative stress with accumulation of malondialdehyde (MDA) concentration as well as reduction of total superoxide dismutase activity of livers in rats [47]. Moreover, candidate biomarkers for geniposide overdose-induced hepatotoxicity were investigated using a label-free quantitative proteomics approach. Five biomarkers in serum were identified, of which aldehyde dehydrogenase 1L1 (ALDH1L1), glycogen phosphorylase (PYGL), glycine N-methyltransferase (GNMT), and alanine-glyoxylate aminotransferase 2 (AGXT2) increased at the beginning of liver injury but then declined gradually; in contrast, *α*-enolase (ENO1) was found to increase consistently in liver injury. Noteworthily, GNMT and PYGL in serum appeared obviously earlier than did ALT and AST, two gold standard biomarkers in liver injury. Hence, these two novel biomarkers could be two indicators to predict early liver injury in the future [51].

## 5. The Main Properties of Geniposide

### 5.1. Anti-inflammatory Activity

Inflammation is not only a self-protection mechanism in the body to guarantee removal of the detrimental stimuli but also a healing process to repair damaged tissue, which is usually caused by various factors, like wound, microbial infection, and myocardial ischemia. However, it would be fetal for the human body if immune cells overproduce cytokines to overwhelm pathogens [52]. Thus, some plant-derived natural components with anti-inflammation effects but without obvious side effects will benefit people a lot.

Currently, geniposide has been proved to possess potent anti-inflammatory action under various pathological conditions. At the beginning, geniposide displayed a favorable anti-inflammation effect on paw edema induced by carrageenan. To be more specific, the edema paw volume gradually increased in the rats without administration of geniposide (up to 3 h). But in the group after injection of extraction from Gardenia fruit at doses of 50, 100, 200, and 400 mg/kg, it exhibited inhibiting effects of 10.2%, 25.9%, 28.6%, and 35.8% in acute paw edema at 3 h. Oral administration of geniposide at a dose of 100 mg/kg contributed to a stronger inhibiting effect of 31.7% at 3 h after edema [5]. However, this study just detected the phenotype of geniposide on paw edema but not explored the further molecular mechanisms.

Lipopolysaccharide (LPS) is the main component in the cell wall of Gram-negative bacteria. Recognizing LPS as well as its signal transduction is an important process of the body's own defense response. LPS is also a vital mechanism that causes endotoxic shock, systemic inflammatory response syndrome, and multiple organ failure. Interleukins (ILs) refer to the interaction of leukocytes or immune cells of lymphokines, which are similar cytokines with hematopoietic growth factor (HGF). The coordination and interaction of IL and HGF contribute to the hematopoietic and immunomodulatory functions. ILs display multiple physiological functions in transmitting information, activating and regulating immune cells, and mediating T and B cell activation, proliferation, and differentiation; in the meantime, they play important roles in the inflammatory response [53–55]. Geniposide remarkably suppressed the production of some inflammatory cytokines including IL-6 and IL-1*β* and tumor necrosis factor-*α* (TNF-*α*) in an *in vitro* model of primary mouse macrophages stimulated by LPS and an *in vivo* lung injury model induced by LPS through blocking the phosphorylation of inhibitory kappa B (I*κ*B*α*) protein, p38, p65, extracellular signal-regulated kinase (ERK), c-Jun N-terminal kinase (JNK), and Toll-like receptor 4 (TLR4) [56, 57]. Meantime, in LPS-induced human umbilical vein endothelial cells (HUVECs), geniposide reduced the level of IL-6 and IL-8 effectively and synchronously blocked HUVEC migration and U937 monocyte adhesion to HUVECs, which was involved in the inactivation of ERK, p38MAPK, and nuclear factor-kappa B (NF-*κ*B) signaling pathways [58]. Geniposide also significantly inhibited LPS-induced nitric oxide (NO), prostaglandin E2 (PGE2), and inflammatory cytokines by downregulating NF-*κ*B, MAPK, and activator protein- (AP-) 1 signaling pathways [59].

**Figure 3:**
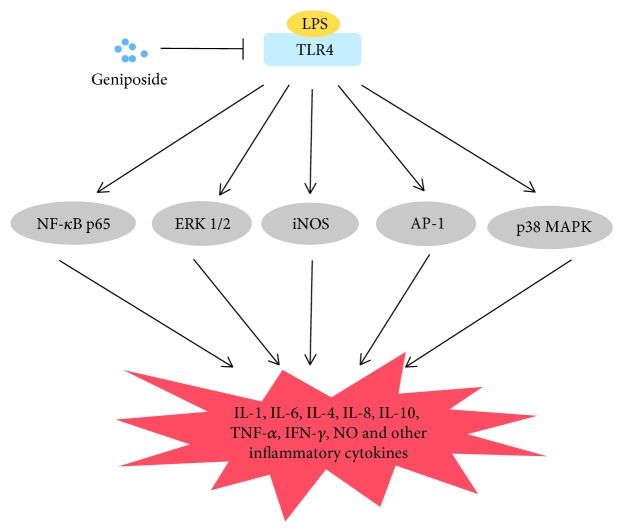
Schematic diagram of mechanisms for anti-inflammatory effects of geniposide. Geniposide inhibits the TLR4-mediated signaling cascades in inflammation induced by LPS. LPS: lipopolysaccharide; TLR4: Toll-like receptor 4; ERK: extracellular signal-regulated kinase; NF-*κ*B: nuclear factor-kappa B; AP-1: activator protein-1; iNOS: inducible nitric oxide synthesis; IL: interleukin; NO: nitric oxide; IFN-*γ*: interferon-*γ*; TNF-*α*: tumor necrosis factor-*α*; MAPK: mitogen-activated protein kinase.

Apart from being against the inflammation induced by LPS, geniposide also displays a favorable anti-inflammatory effect in the process of ischemia/reperfusion (I/R) injury in cerebral tissue through the activation of TLR4. To our knowledge, once the microglia are activated after I/R, it will become hypertrophic and proliferate rapidly, then migrate to the inflammatory region where they will produce a mass of proinflammatory and neurotoxic mediators, subsequently causing neuron injury [60]. Geniposide protected neurons from I/R injury in rats and mitigate oxygen-glucose deprivation-induced activation of microglial cells through decreasing various inflammatory cytokines, such as IL-1*β*, TNF-*α*, IL-6, IL-8, and IL-10, and blocking upstream TLR4, NF-*κ*B, and MAPK pathways [61]. TLR4 is a type of protein involving the detection of endotoxins (such as LPS) produced by Gram-negative bacteria and signals through MyD88, subsequently giving rise to the downstream activation of NF-*κ*B and MAPK signaling cascade [62–64]. Therefore, TLR4 could be a promising drug target of geniposide to fight against inflammation in a wide range of cell types. A schematic diagram of mechanisms for the anti-inflammatory effect of geniposide is shown in Figure 3. Additionally, geniposide owned *in vitro and in vivo* anti-inflammation and antiviral action in the infection of pandemic A/Jiangsu/1/2009 (H1N1) influenza virus. Geniposide (20 mg/kg/day) had similar clinical effects with the neuraminidase inhibitor (peramivir) (30 mg/kg/day) in pandemic A/Jiangsu/1/2009 (H1N1) influenza virus-induced Madin-Darby canine kidney (MDCK) cell injury by suppressing IL-4, IL-6 and IL-10, TNF-*α*, and interferon-*γ* (IFN-*γ*) [65].

In fact, genipin similarly possesses a topical, potent, anti-inflammatory effect. Genipin was observed to exhibit higher inhibitory activity on inflammation (49.1% vs. 31.7%) at a lower dose (50 mg/kg) than geniposide at a higher dose (100 mg/kg) in the rat paw edema model [5]. Also, genipin in a concentration-dependent manner suppressed the production of NO and inducible nitric oxide synthesis (iNOS) in murine macrophage cells stimulated by LPS and IFN-*γ*, which helped to block NF-*κ*B activation [6].

### 5.2. Antioxidant Activity

**Figure 4:**
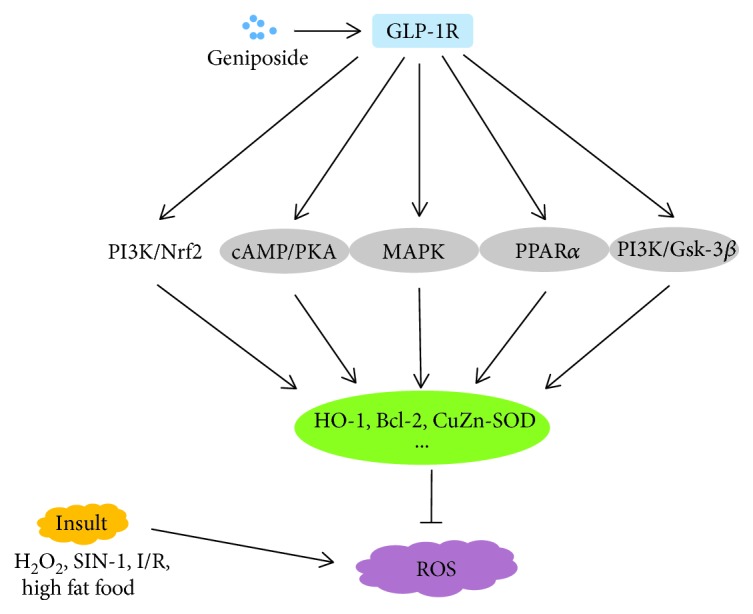
Schematic diagram of mechanisms for antioxidant activity of geniposide. Geniposide activates the GLP-1R-mediated signaling cascades in oxidative stress induced by H_2_O_2_, SIN-1, I/R, and high-fat food. ROS: reactive oxygen species; GLP-1R: glucagon-like peptide-1 receptor; CuZn-SOD: copper- and zinc-containing superoxide dismutase; SIN-1: 3-morpholinosydnonimine hydrochloride; PKA: protein kinase A; MAPK: mitogen-activated protein kinase; PPAR*α*: peroxisome proliferator-activated receptor-*α*; PI3K: phosphatidylinositol-3 kinase; HO-1: heme oxygenase-1; Nrf2: nuclear factor-E2-related factor 2.

Oxidative stress generally occurs under an imbalanced redox status which encompasses either dysfunction of the antioxidant system or excessive production of reactive oxygen species (ROS). Dysregulated oxidative stress is appearing as an obvious causal factor in aging body and some age-related diseases [66, 67]. Cumulative evidence discloses that geniposide could upregulate endogenous antioxidative enzymes to be a promising strategy for delaying the process of cell injury (Figure 4).

GLP-1 receptor is a member of the class B1 family of seven-transmembrane-spanning receptors, belonging to heterotrimeric G protein-coupled receptors (GPCRs) [68]. The signal transduction of the GLP-1 receptor is mainly mediated through the calcium and cyclic AMP (cAMP)/protein kinase A (PKA) signaling cascades [69]. The mechanisms associated with the neuroprotection of geniposide in PC12 cells against oxidative damage are involved in three signaling pathways. First, geniposide increased the expression of heme oxygenase-1 (HO-1) via the cAMP/PKA/cAMP response element-binding protein (CREB) signaling pathway [70]. Second, geniposide upregulated the expression of some antioxidative proteins involving HO-1 and Bcl-2 through activating the transcription factor of p90RSK via the MAPK signaling pathway [71]. Third, geniposide enhanced the phosphorylation of Akt473, Akt308, GSK-3*β*, and PDK1 in the phosphatidylinositol-3 kinase (PI3K) signaling pathway [72]. Furthermore, in Alzheimer's disease mice, geniposide improved learning and memory ability decreased by reducing oxidative stress as well as mitochondrial dysfunction via alleviating the mitochondrial oxidative damage and elevating the activity of cytochrome c oxidase and the mitochondrial membrane potential [68].

In addition, geniposide and genipin also conferred significant hepatoprotection by decreasing oxidative stress. On the one hand, geniposide potentiated the expression of HO-1 and attenuated cytochrome c protein, tBid expression, and caspase-3 activity [38]. On the other hand, geniposide also protected against hepatic steatosis in high fat diet-induced rats by its antioxidant actions and its regulation on adipocytokine release. In detail, geniposide increased the expression of peroxisome proliferator-activated receptor-*α* (PPAR*α*) in addition to suppressing the expression of CYP2E1, giving rise to elevated superoxide dismutase and reduced MDA in liver [38]. Also, geniposide upregulated the level of some main antioxidant enzymes including glutathione peroxidase (GPx), hepatic lipid peroxidation (LPO), glutathione-*S*-transferase (GST), glutathione (GSH), and copper- and zinc-containing superoxide dismutase (CuZn-SOD) and thus protected against alcohol-induced oxidative stress injury in liver [9].

## 6. Preclinical Evidence for the Therapeutic Potential of Geniposide on Cardiovascular Disease and DM

Accumulating evidence demonstrated that geniposide exerted potential therapeutic benefits through modulating various genes and proteins associated with oxidative stress and inflammation. In this section, we mainly focused on their therapeutic potential in cardiovascular disease, and the details are summarized in Table 3.

### 6.1. Cardiovascular Disease

#### 6.1.1. Cardiac Fibrosis

**Figure 5:**
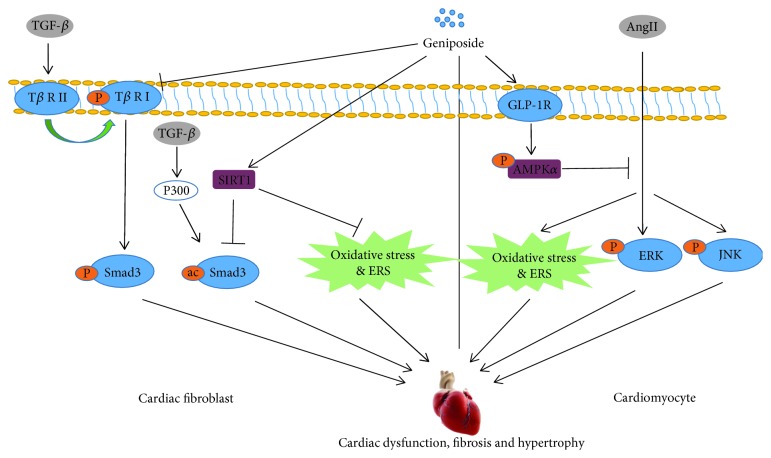
Schematic diagram of mechanisms in cardiac fibrosis and cardiac hypertrophy of geniposide. TGF-*β*: transforming growth factor-*β*; AngII: angiotensin II; T*β* R: TGF-*β* receptor; ERS: endoplasmic reticulum stress; GLP-1R: glucagon-like peptide-1 receptor; AMPK*α*: AMP-activated protein kinase *α*; ERK: extracellular signal-regulated kinase; JNK: c-Jun NH2-terminal kinase; P: phosphorylated; ac: acetylated.

Cardiac fibrosis is a common pathological alteration in myocardial infarction (MI), hypertension, dilated cardiomyopathy, and DM [73]. In the development of cardiac fibrosis, overproduction and deposition of collagen and extracellular matrix (ECM) will increase the stiffness of vessel, meanwhile deteriorating diastolic function, contributing to heart failure eventually [74]. Studies demonstrated that oxidative stress and ERS display close and critical relationships with cardiac fibrosis. On the one hand, cardiac oxidative stress could enhance the ability of collagen synthesis and inhibit collagen degradation in cardiac fibroblasts [75]. On the other hand, oxidative stress triggers accumulation of ECM in the heart of rats with diabetic cardiomyopathy by activating genes implicated with cardiac fibrosis, like TGF-*β*, fibronectin, and connective tissue growth factor (CTGF), apart from activating the NF-*κ*B signaling pathway [76]. Sirtuin 1 deacetylase (SIRT1) is one of the mammalian homologs of the yeast silent information regulator 2 protein, which in essence is a nicotinamide adenine dinucleotide NAD(+)-dependent deacetylase [77]. A potent agonist of SIRT1, resveratrol, was found to inhibit the production of ROS in cardiomyocytes by activating SIRT1 and enhancing mitochondrial biogenesis [78]. Our previous study unveiled that geniposide could alleviate isoprenaline-induced cardiac dysfunction and fibrosis in the mouse model with cardiac fibrosis. An *in vitro* experiment disclosed that geniposide also suppressed the transformation of cardiac fibroblasts into myofibroblasts stimulated by TGF-*β*. The mechanism of anti-cardiac fibrosis from geniposide is associated with the inhibition of the acetylated- (ac-) Smad3 pathway, oxidative stress, and ERS in a SIRT1-dependent manner; meanwhile, geniposide could also reduce phosphorylated- (p-) Smad3 level by inactivating the type II TGF-*β* receptor independent of SIRT1 activation [78] (Figure 5).

#### 6.1.2. Cardiac Hypertrophy

Cardiac hypertrophy is the response of the heart to multifarious intrinsic and extrinsic stimuli imposing enhanced biomechanical stress, which can deteriorate to heart failure and eventually cause high mortality [79]. One of the mechanisms contributing to cardiac hypertrophy is oxidative stress and ERS. Once oxidative stress and ERS in the myocardium are initiated, branches of certain protein responses will elevate ROS and bring about apoptosis, resulting in the process of cardiac hypertrophy [80]. AMPK*α* as a vital regulator of cardiac homeostasis can block the synthesis of proteins by antagonizing mTOR kinase, which has been disclosed to act as an intrinsic suppressor in oxidative stress and ERS [81]. Additionally, activating AMPK*α* also prevented the heart from I/R injury via the inhibition of oxidative stress ad ERS [82]. Our previous study uncovered that, in the mouse model with transverse aortic constriction (TAC), geniposide remarkably blocked the hypertrophic response by increasing the left ventricular internal diastolic diameter and fractional shortening and decreasing the ejection fraction. Activation of AMPK*α* and suppression of mTOR, ERK, and ERS were observed in hypertrophic hearts and angiotensin II- (AngII-) induced H9c2 cardiomyocytes that were treated with geniposide. However, all of these alterations were counteracted after AMPK*α* was blocked by compound C or the GLP-1 receptor was knocked down, implying that the protective roles of geniposide on cardiac hypertrophy were mediated by the GLP-1 receptor/AMPK*α* signaling pathway [17] (Figure 5).

#### 6.1.3. Myocardium I/R

Myocardium I/R injury is one main cause of morbidity and mortality implicated with ischemia heart disease, the typical pathological alterations of which were cardiomyocyte apoptosis and overproduction of ROS [83]. It is universally recognized that unbalanced and high steady-state levels of ROS and nitrogen species (RNS) are responsible for cytotoxicity in the heart, which gives rise to contractile dysfunction and cardiomyocyte death [84]. Ischemia is featured by a virtual lack of O_2_ and other critical substrates. Long-term ischemia could accumulate various metabolites including lactate, which further induces a cascade of events involving excess ROS/RNS generation, disruption of Ca^2+^ homeostasis, and loss of nucleotide homeostasis [85]. Mitochondria not only are the main enzymatic source of ROS but also were major target organelles for ROS-mediated damage in cells [86]. In MI, excess ROS could trigger mitochondrial DNA (mtDNA) damage, which further gives rise to dysregulation of respiratory chain complex enzymes and mtDNA-encoded genes, eventually resulting in the occurrence of cardiac remodelling and heart failure [87].

The previous study showed that geniposide was able to block the oxidative stress and apoptosis of H9c2 cells challenged with hypoxia/reoxygenation by improving mitochondrial function, as shown by improving mitochondrial morphological alterations, relieving mitochondrial oxidative stress, decreasing mitochondrial calcium overload, and maintaining mitochondrial membrane potential. These antioxidative effects of geniposide on mitochondria in I/R injury were partly due to activation of the GLP-1 receptor and downstream PI3K/Akt signaling pathway [10].

#### 6.1.4. Obesity-Related Cardiac Injury

Obesity, a risk factor for cardiovascular disease, triggers structural and functional alterations in the myocardium [88]. Long-term obesity could predispose an individual to various cardiac complications, involving cardiac dysfunction, cardiac energy metabolism disturbance and remodelling, and even heart failure [89]. The driving forces in obesity-related cardiac injury involve an excess supply of saturated fatty acids, fibrosis, hypertrophy, myocardial inflammation, loss of cardiomyocytes, and epigenetics [90–92]. Our previous study demonstrated that oral administration of geniposide could inhibit obesity and obesity-induced cardiac injury. To be more specific, we found that geniposide alleviated myocardial inflammation in high-fat diet- (HFD-) induced mice in a SIRT1-dependent manner; meanwhile, geniposide combated cardiomyocyte apoptosis in an AMPK*α*-dependent manner. In particular, the activation of SIRT1 and AMPK*α* in the myocardium was mediated by the GLP-1R [92]. Our study hints that geniposide possesses the potential to be a clinical candidate for the treatment of obesity and obesity-related cardiac injury.

#### 6.1.5. Atherosclerosis

**Figure 6:**
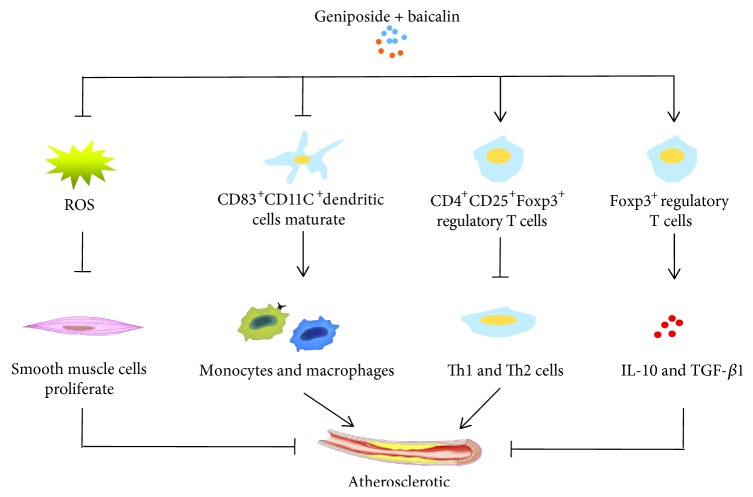
Schematic diagram of mechanisms of geniposide in atherosclerotic. ROS: reactive oxide species; Th cells: T helper cells; IL: interleukin.

Atherosclerosis is featured by vascular oxidative stress, inflammation, endothelial dysfunction, and formation of lipids in the vessel intima, which is also followed by proliferation and migration of vascular smooth muscle cells, as well as endothelial activation initiated by various inflammatory molecules, and finally causes the buildup of lipid plaque [93]. Studies demonstrated that geniposide combined with another herb, baicalin, could obviously attenuate the formation of atherosclerotic plaque in high-lipid diet-treated mice through increasing the proliferation of smooth muscle cells and suppressing oxidative stress and inflammation, the underlying mechanisms of which may be involved in inhibiting Wnt1 and enhancing the ratio of Wnt1/dickkopf-related protein 1 (DKK1) [94]. Dendritic cells, a type of professional antigen-presenting cells implicated with innate and adaptive immune responses, are also proved to modulate the immune status in atherosclerosis [95, 96]. During the process of atherosclerosis, a great many dendritic cells are discovered inside the adventitial layer of blood vessels and atherosclerotic plaques [97]. On the contrary, the absence of dendritic cells or inhibition of the maturation of dendritic cells could alleviate atherosclerotic lesions by reducing the accumulation of monocytes and macrophages [98, 99]. In ApoE−/− mice fed a high-cholesterol diet, either geniposide or baicalin or a combined administration of the two drugs can regress the process of atherosclerosis through lowering the concentration of low-density lipoprotein (LDL) and total cholesterol (TC) in serum. Interestingly, either geniposide or baicalin alone could reduce the number of CD83-expressing and CD11C dendritic cells in the bone marrow, but the CD83 expression in atherosclerotic lesions was not decreased. However, when combining geniposide and baicalin, the expression, maturation, mobilization, and infiltration of CD83 and CD11C in both atherosclerotic lesions and bone marrow were inhibited [99]. We speculate that the drug-drug interactions may improve the pharmaceutical effects of geniposide on atherosclerosis, but the specific mechanism needs further exploring. Apart from dendritic cells, other immunocytes such as Th1 and Th2 cells are also captured in atherosclerotic plaque, which are highly associated with the process of atherosclerosis. It has been reported that Th2 cells contribute to a beneficial response while Th1 cells have the opposite effects in atherosclerosis [100]. One of the subtypes from T helper cells, CD4^+^CD25^+^Foxp3^+^ regulatory T cells, were identified to exclusively repress both Th1 and Th2 cells and therefore inhibited the development of atherosclerotic lesions [101]. Furthermore, Treg cells that specifically express the forkhead/winged helix transcription factor (Foxp3) display potential anti-inflammatory effects by secreting IL-10 and TGF-*β*1 [102]. Similarly, pretreatment with geniposide or baicalin helped the ApoE-/- mice with high-cholesterol diet produce more splenic Treg cells, TGF-*β*1, and IL-10, in addition to notably decreasing serum LDL and TC. At the same time, the levels of Foxp3 and Foxp3^+^ regulatory T cells in atherosclerotic lesions were significantly enhanced by geniposide and baicalin [103]. Taken together, the three studies above demonstrate that geniposide is both an immunoregulatory agent and a lipid-regulation agent in protecting against atherosclerosis and combination with baicalin will improve the efficacy of geniposide (Figure 6).

#### 6.1.6. Ischemic Stroke

Ischemic stroke, accounting for about 85% of all stroke cases, correlates with high morbidity and mortality globally [104]. Ischemic stroke will further deteriorate to cerebral I/R injury by sudden recovery of the blood supply unless managed appropriately, finally causing cerebral microcirculatory damage and death of neurons [105]. Autophagy activation, ERS, and oxidative stress play significant roles in the development of cerebral I/R injury [106, 107]. Treatment with a combination of geniposide and tauroursodeoxycholic acid could counteract the elevation in cellular ROS levels, the activation of oxygen-glucose deprivation-reoxygenation- (OGD/R-) induced ERS, and autophagy in SH-SY5Y cells with hypoxia-reoxygenation injury [108]. On the other hand, inflammatory events that occur between the blood-endothelium interface of the cerebral capillaries also underlie the resultant ischemic tissue damage [109]. However, these inflammatory events in cerebral ischemia could block geniposide via activating the P2Y14 receptor. In the cellular model of OGD-induced cerebral ischemia, the level of P2Y14 receptor and its downstream signaling pathways involving RAF-1, MEK1/2, and ERK1/2 were all downregulated by geniposide. At the same time, the production of IL-1*β*, IL-8, and MCP-1 in brain microvascular endothelial cells (BMECs) were also suppressed. These results indicated that anti-inflammatory and antioxidative effects of geniposide in cerebral ischemia depend on the expression of the P2Y14 receptor and its downstream ERK1/2 signaling pathways [110].

Calcium-permeable ionotropic N-methyl-D-aspartate receptor- (NMDAR-) mediated Ca^2+^ overload, glutamatergic overexcitation, and oxidative stress are believed to be vital factors in regulating the development of ischemic stroke [111]. Assuming that NMDAR overexcitation could disturb the equilibrium between inhibition and excitation in I/R injury, restoring this balance by intervening in neural transmission in the post-ischemic brain is likely to be a selective and useful strategy [112, 113], GluN2A and GluN2B are the major subtypes of NMDAR in the hippocampus, which are, respectively, responsible for neuronal survival and death following I/R [114]. Notably, geniposide could decrease the infarct volume in transient middle cerebral artery occlusion (tMCAO) rats in a dose-dependent manner. A medium level of geniposide applied *in vivo* effectively prevented post-stroke injury by regulating blood brain barrier leakage/hemorrhage and neuronal loss/apoptosis, meanwhile enhancing the expression of GluN2A, but not GluN2B. However, when applying the GluN2A antagonist, NVP, the therapeutic effects in MCAO rats could be blocked, implying that GluN2A is a potential target of geniposide. Whereafter, activation of GluN2A led to a remarkable increase in network excitation, which further activated prosurvival signals involving pAKT, pERK, and PSD-95 in ischemia. In brief, the GluN2A/AKT/ERK axis was essential for the neuroprotective effects of geniposide in post-ischemic neurovascular injury [115].

#### 6.1.7. Hypertension

Moreover, the antihypertension effects of *Gardenia jasminoides* were also investigated in the L-NG-nitroarginine- (L-NNA-) induced hypertension mouse model. The *Gardenia jasminoides*-treated mice displayed lower diastolic blood pressure, systolic blood pressure, and mean blood pressure compared with the hypertensive mice without administration of *Gardenia jasminoides*. Molecular biological assays showed that the level of NO in multiple tissues including the serum, heart, kidney, stomach, and liver of *Gardenia jasminoides*-treated mice were higher, but the malondialdehyde contents were lower. In the hypertensive mice treated with *Gardenia jasminoides*, a higher mRNA expression of neuronal NOS, HO-1, Bax, caspase-3, -8, and -9, and endothelial NOS and a lower mRNA expression of receptor activity-modifying protein (RAMP), adrenomedullin, IL-1*β*, TNF-*α*, Bcl-2, inducible NOS, monocyte chemoattractant protein-1 (MCP-1), NF-*κ*B, and matrix metalloproteinase- (MMP-) 2 and -9 were found, indicating that the antihypertension effects of *Gardenia jasminoides* may be implicated with its antiapoptosis and antioxidative stress. But the active component in *Gardenia jasminoides* (geniposide, genipin, crocin 1, and crocin 2) for antihypertension is not yet clear [116].

### 6.2. Diabetes Mellitus

**Figure 7:**
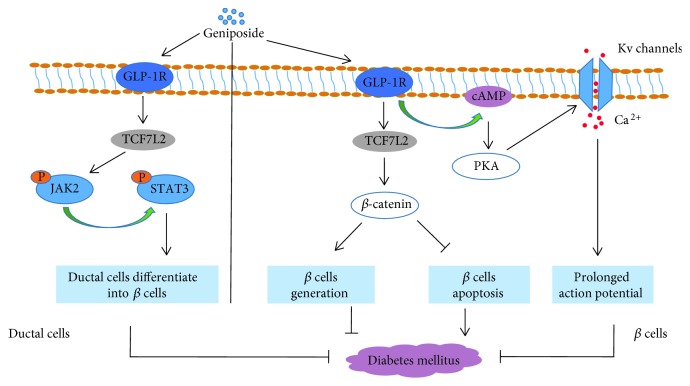
Schematic diagram of mechanisms of geniposide in diabetes mellitus. TCF7L2: T-cell factor 7-like 2; GLP-1R: glucagon-like peptide-1 receptor; PKA: protein kinase A; cAMP: cyclic adenosine monophosphate; Kv channels: voltage-gated K channels; JAK2: tyrosine kinase 2; P: phosphorylated.

Diabetes mellitus (DM) is a serious, chronic disease which occurs either when the body does not use the insulin produced by itself effectively meaning insulin resistance (type 2 DM) or when the pancreas cannot produce sufficient insulin meaning insulin deficiency (type 1 DM). Over the past few decades, both the number of the prevalence and cases of DM have been steadily growing [117]. Geniposide displays potent effects against DM through multiple mechanisms (Figure 7).

#### 6.2.1. Type 2 DM

Previous study showed that the metabolic syndrome increased the risk for DM independently of other risk factors. With the accumulation of visceral fat, many symptoms of metabolic syndrome such as abnormal glucose/lipid metabolism and insulin resistance are induced, which eventually give rise to the onset of ischemic cerebrovascular disease [118]. In spontaneously obese type 2 DM TSOD mice, geniposide reduced the body weight and visceral fat accumulation and alleviated abnormal lipid metabolism as well as intrahepatic lipid accumulation. Meanwhile, it also exerted good therapeutic effects on hyperinsulinemia and abnormal glucose tolerance. To further explore the direct function of geniposide on the liver, genipin was used in free fatty acid-treated mice. Genipin not merely blocked the accumulation of intracellular lipid which resulted from the free fatty acid treatment but also enhanced the intracellular fatty acid oxidation-related ge HepG2 fatty liver modene (PPAR*α*) expression at the same time. Taken together, these results demonstrated that geniposide possessed antiobesity and antioxidative effects, insulin resistance-alleviating effects, and abnormal lipid metabolism-alleviating effects, but it is genipin that showed a direct effect on liver through enhancing the expression of PPAR*α* [16].

Autophagy is an intracellular process, which could sequester damaged or senescent organelles and proteins in autophagosomes to recycle their degradation products [119, 120]. Insulin and intracellular molecules involving mTOR can inhibit the process of autophagy, whereas glucagon as a type of counterregulatory hormone of insulin can activate autophagy, implying that autophagy may be implicated with the natural history of DM via its involvement in either organelle function or hormone activity [121]. As a key complex connected with autophagosomal membranes, p62/SQSTM1 (P62) could swallow cytoplasmic content for subsequent degradation. Hyperglycemia enhances the p62/PKC interaction and autophagy, mediating the activation of NF-*κ*B and increasing the expression of NADPH oxidase 4 [122]. In addition, glucose transporter 4 (GLUT-4) is a vital medium in transport of extracellular glucose to insulin-sensitive cells, the translocation of which to the insulin-sensitive cell membrane is modulated by insulin. In a HepG2 cell model of insulin resistance, geniposide treatment initially potentiated the expression of GLUT-4, whereafter it decreased its expression in time to its nadir at 8 h. In the meantime, the level of autophagy was significantly enhanced by geniposide through modulating the expression of LC3 and P62. Also, the NF-*κ*B signaling pathway was blocked by both the direct effect of geniposide and the activation of autophagy, resulting in alleviation of insulin resistance in HepG2 cells eventually [123].

As a result of loss of *β*-cell mass or defects in insulin secretion, type 2 DM is also connected with the increased level of *β*-cell apoptosis, which makes pancreatic *β* cells cannot compensate for peripheral insulin resistance [124]. The emerging evidence uncovers that lipotoxicity may undertake an essential role in pancreatic *β*-cell apoptosis and oxidative stress in type 2 DM. A high level of plasma free fatty acids is cytotoxic to pancreatic *β* cells, which activates apoptotic and oxidative signaling pathways to initiate mitochondrial perturbation and causes oxidative stress [125]. Hence, assuming that a drug can preserve *β*-cell mass by inhibiting lipotoxicity, it may be a promising candidate against the development of DM. Liu et al. found that pretreatment of pancreatic INS-1 cells with geniposide for 7 h relieved *β*-cell apoptosis induced by palmitate, but at 18 h, this effect disappeared. Additionally, in palmitate-treated INS-1 cells, geniposide also attenuated the impairment of GLP-1R by increasing the phosphorylation of Foxo1 and AKT and enhanced the expression of PDX-1, indicating that GLP-1R was a key part in alleviating lipotoxicity-induced *β*-cell apoptosis by geniposide [126]. When pancreatic *β*-cells are chronically exposed to high glucose concentration for an extended time, glucose-stimulated insulin secretion in *β*-cells would be impaired and *β*-cell apoptosis also increased, which is so-called glucotoxicity-related apoptosis [127]. However, the glucose-induced impairment of insulin secretion and injury of the pancreas islet could be obviously reverted by the inclusion of 1 or 10 *μ*M of geniposide. Induction of AMPK by geniposide was involved in promoting the use of nutrient stores for energy production and antagonizing pancreatic *β*-cell injury [128]. Intriguingly, the effects of geniposide on acute glucose-stimulated insulin secretion in INS-1 cells depended on the concentration of glucose. Geniposide could significantly enhance insulin secretion in response to the acute stimulation of low or moderately high concentrations of glucose and promoted glucose uptake as well as the production of intracellular ATP. But in INS-1 cells stimulated acutely by high concentrations of glucose, these protective effects disappeared. This may be because of the capacity to preserve pancreatic *β*-cells from exhaustion and damage caused by prolonged and oversecretion of insulin as well as glucotoxicity under high glucose burden [129].

Apart from inhibiting *β*-cell apoptosis, another therapeutic strategy for DM is to promote new *β*-cell formation. The study showed that proliferation of preexisting *β*-cells can generate new *β*-cells [130]. Hence, facultative progenitors in regenerating pancreatic ducts could be a promising source of *β*-cells [131]. By activating T-cell factor 7-like 2 (TCF7L2), a crucial transcription factor in Wnt/*β*-catenin signaling and having vital roles in *β*-cell survival and regeneration, geniposide remarkably increased *β*-cell survival. Geniposide could trigger duct cell differentiation through enhancing TCF7L2 expression and activating the JAK2/STAT3 pathway in exocrine cells isolated from the mouse pancreas. The two mechanisms of geniposide promoted its regeneration in cultured mouse islets after challenge with diabetic stimuli [132].

#### 6.2.2. Type 1 DM

The role of geniposide in type 1 DM was also investigated. In rat pancreatic islets, geniposide enhanced insulin secretion via activating the GLP-1R as well as its downstream adenylyl cyclase (AC)/cAMP signaling pathway. However, inhibition of PKA blocked the insulinotropic effect from geniposide. On the other hand, geniposide also suppressed voltage-dependent potassium (Kv) channels (Ca^2+^ channels) and prolonged the action potential duration, but this effect could be reversed by inhibiting PKA or GLP-1R. Collectively, these results demonstrated that geniposide acutely improved the ability of insulin secretion in pancreatic *β*-cells through activating GLP-1R/cAMP signaling and Ca^2+^ channels were also involved in this process [133]. Further research unveiled that geniposide in a time-dependent manner induced phosphorylation of acetyl-CoA carboxylase (ACC), a marker of AMPK activity and enhanced glucose uptake, energy metabolism, and insulin secretion in rat pancreatic INS-1 cells; however, these effects were remedied by compound C (an AMPK inhibitor) [133].

#### 6.2.3. Diabetic Nephropathy

Diabetic nephropathy is one of the most common complications of DM and is also a major factor causing end-stage renal disease, the pathogenesis of which is implicated with immunologic and inflammatory response [134]. Intragastric administration of geniposide ameliorated structural and functional abnormalities of the kidney by suppressing NF-*κ*B-mediated inflammation response in diabetic rats induced by streptozotocin [135].

## 7. Future Perspective of Geniposide

Over the last several decades, a great number of studies in animal and cell models have uncovered the potential of geniposide for the treatment of a vast array of diseases, ranging from neurological diseases to cardiovascular diseases and DM. However, we still face the following problems to be solved urgently in terms of the clinical application of geniposide. For instance, at present, the studies on geniposide only stay at animal and cell levels and have not been carried out in the human body. Hence, further clinical studies are imperative to determining a conclusive role for geniposide in human therapeutics. Additionally, although the pharmacokinetics of geniposide has made a lot of progress, we need more details on its transformation, monitoring, and metabolism so that its efficacy and safety are guaranteed. Firstly, we need to employ some methods to increase the transformation rate of geniposide into genipin. Yang and his colleagues [136] investigated the hydrolysis of geniposide to genipin by *β*-glucosidase immobilized by the crosslinking-embedding in an aqueous-organic two-phase system. The optimum reaction pH value of immobilized and free *β*-glucosidase was 4.5 while the optimum reaction temperature was 55°C and 50°C, respectively. Also, the immobilization decreased the effect of pH variation on the activity of *β*-glucosidase and enhanced its heat resistance. Secondly, natural medicines often possess relatively low active ingredients, the concentration of which is difficult to detect after entering the peripheral blood. Therefore, to further optimize the monitoring program, we had better take advantage of a variety of methods and combine multiple indicators. Thirdly, few studies focus on the metabolites of geniposide currently; hence, we should further evaluate the structure of its metabolites, metabolic pathways, and pharmacological effects in order to elucidate the true molecular basis of pharmacological actions of geniposide. Fourthly, in order to acquire a relatively high geniposide content with low cost, more plants as well as their parts need to be detected to find the optimal candidate for geniposide preparation. All of these mentioned above will be of great importance for its new drug research and clinical rational use.

## 8. Conclusion

In this review, we uncover the pharmacokinetics and preclinical values of geniposide, which might be beneficial and referential for clinical applications and future investigations. We discover that the pharmacokinetics of geniposide is complicated and it can be decomposed into multiple metabolites in vivo. Geniposide predominately exists in the liver and kidney and is excreted in the original form via the kidney. Furthermore, geniposide possesses a great body of pharmacological effects involving antioxidative stress, anti-inflammation, and antiapoptosis. Owing to these favorable effects, geniposide serves as a weapon against various disorders, especially cardiovascular disease and DM. Although numerous investigations are exploring the preclinical values of geniposide, we assume that geniposide has more possible targets and molecular pathways in disease treatment, which demands further investigation. In particular, the use of these natural antioxidants like geniposide should not be indiscriminate in that it still lacks clinical proofs at present. Hence, more empirical data pertaining to improvement in clinical application, safety, and efficacy of geniposide are demanded before geniposide could be translated from bench to bedside. Nevertheless, geniposide does seem to be on behalf of the light at the end of the tunnel as a supportive and ideal remedy for allopathic treatment considering its wide range of purported efficacy and the relatively lesser incidence of adverse reactions. Sincerely, we hope this review might provide some proofs for clinical applications and further studies in the future.

## Figures and Tables

**Table 1:** Amounts of genipin transformed from geniposide by incubation with 24 strains of human intestinal bacteria. Each tube contained 30 mg geniposide.

Strain	Genipin (mg/tube)	Strain	Genipin (mg/tube)
*Klebsiella pneumoniae* ATCC 13883	3.8	*Bifidobacterium adolescentis*	1.2
*Bacteroides fragilis ssp. thetaotus*	2.0	*Lactobacillus acidophilus* ATCC 4356	6.6
*L. brevis* II-46	1.4	*B. bifidum*	0.3
*L. fermentum* ATCC 9338	6.9	*B. breve* S-2 KZ 1287	2.4
*B. pseudolongum* PNC-2-9-G	1.8	*L. xylosus* ATCC 155775	6.2
*L. plantarum* ATCC 14917	5.3	*B. longum*	0.9
*Clostridium butyricum*	0.7	*Peptostreptococcus anaerobius*	1.4
*C. innocuum* KZ-633	1.2	*Proteus mirabilis* S2	3.3
*C. perfringens* To-23	0.3	*C. innocuum* ES 24-06	0.3
*P. intermedium*	0.9	Fusobacterium nucleatum	3.8
*Escherichia coli* 0-127	5.3	*Ruminococcus sp.* PO1-3	2.9
*Streptococcus faecalis* II-136	2.5	*Veillonella parvula ssp. parvula*	0.4

In the meantime, each individual bacterial suspension was incubated for 24 hours anaerobically in 100 mM phosphate buffer (pH 7.3) at 37°C.

**Table 2:** The distribution of geniposide and its metabolites in rats with adjuvant arthritis.

	Plasma	Spleen	Urine	Liver	Synovium	Mesenteric lymph node
Geniposide	+	+	+	+	+	+
G1	+	—	+	—	—	—
G2	+	+	+	—	—	+
G3	—	+	—	—	—	—
G4	—	+	+	—	—	—

G1, G2, G3, and G4 are four major metabolites of geniposide.

**Table 3:** Preclinical evidence of therapeutic potential from geniposide.

System	Disorder	Model	Dose	Administration route	Pharmacological action	Reference
Endocrine system	Type 2 DM	Spontaneously obese Type 2 diabetic TSOD mice	0.1% and 0.3%	Intragastric administration	Reducing the body weight and visceral fat accumulation and alleviating abnormal lipid metabolism and intrahepatic lipid accumulation	[16]
	Type 2 DM	Free fatty acid-treated HepG2 cells	10, 50, 100 *μ*M	Incubation	Blocking the accumulation of intracellular lipid resulted from the free fatty acid treatment and enhanced the PPAR*α* expression	[16]
	Type 2 DM	HepG2 cell model of insulin resistance	15.63-125 mg/L	Incubation	Promoting autophagy and inhibiting insulin resistance in the HepG2 cells, which may be associated with the dynamic regulation of the P62/NF-*κ*B/GLUT-4 pathway	[123]
	Type 2 DM	Palmitate-treated INS-1 cells	1, 10, 100 *μ*M	Incubation	Relieving *β*-cell apoptosis via activation of the GLP-1 receptor	[132]
	Type 2 DM	Cultured mouse islets after challenge with diabetic stimuli	20 *μ*M	Incubation	Promoting *β* regeneration and survival via regulating *β*-catenin/TCF7L2 pathway	[132]
	Type 2 DM	HepG2 cells	1, 10, 100 *μ*M	Incubation	Suppressing hepatic glucose production via AMPK signaling pathway	[137]
	Type 1or 2 DM	High glucose-induced glucotoxic insulinoma cells	10 *μ*M	Incubation	Improving *β*-cell function and increasing the proliferation of *β*-cells exposed to prolonged hyperglycemia	[128]
	Type 1 DM	Rat pancreatic islets	10 *μ*M	Incubation	Stimulating insulin secretion in pancreatic *β*-cells by regulating GLP-1 receptor/cAMP signaling and Ca^2+^ channels	[133]
	Type 1 DM	Rat pancreatic INS-1 cells	10 *μ*M	Incubation	Enhancing glucose uptake via activating AMPK in pancreatic *β* cells	[138]
	Diabetic nephropathy	Diabetic rats induced by streptozotocin	50, 100 mg/kg/d	Intragastric administration	Suppressing NF-*κ*B mediated inflammation response	[135]

Cardiovascular system	Myocardium I/R	Hypoxia/reoxygenation-induced H9c2 Cells	10, 20, 40, 80 *μ*M	Incubation	Enhancing mitochondrial function via the GLP-1 receptor mediated the PI3K/Akt signaling pathway	[10]
	Atherosclerosis	ApoE^−/−^ mice fed a high-cholesterol diet	100 mg/kg/d	Oral administration	Increasing proliferation of smooth muscle cells and suppressing inflammation	[94]
	Atherosclerosis	ApoE^−/−^ mice fed a high-cholesterol diet	100 mg/kg/d	Oral administration	Decreasing the dendritic cells numbers and inhibiting dendritic cell maturation in bone marrow and infiltration into lesions	[99]
	Atherosclerosis	ApoE^−/−^ mice fed a high-cholesterol diet	100 mg/kg/d	Oral administration	Regulating lipid and promoting the number and function of Treg cells	[103]
	Cardiac hypertrophy	Mice with transverse aortic constriction	50 mg/kg/d	Oral administration	Activating the GLP-1 receptor/AMPK*α* pathway, inhibiting ERS and oxidative stress	[17]
	Cardiac fibrosis	Mice induced by isoprenaline	50 mg/kg/d	Oral administration	Suppressing oxidative stress, ERS, and acetylated Smad3 in a SIRT1-dependent manner and inhibiting the phosphorylated-Samd3 pathway independent of SIRT1 activation	[78]
	Obesity-related cardiac injury	Mice induced by high-fat food	50 mg/kg/d	Oral administration	Alleviating inflammation in a SIRT1-dependent manner and inhibiting cardiomyocyte apoptosis in a AMPK*α*-dependent manner	[92]
	Cerebral I/R injury	OGD/R-induced SH-SY5Y cells	10 *μ*M	Incubation	Inhibition of endoplasmic reticulum stress and autophagy	[108]
	Ischemic stroke.	tMCAO rats	75 mg/kg/d	Intraperitoneal injections	Protecting neurons against post-ischemic neurovascular injury through the activation of GluN2A/AKT/ERK pathways	[115]
